# Phase II study of UFT with leucovorin and irinotecan (TEGAFIRI): first-line therapy for metastatic colorectal cancer

**DOI:** 10.1038/sj.bjc.6603889

**Published:** 2007-07-17

**Authors:** J-P Delord, J Bennouna, P Artru, H Perrier, F Husseini, F Desseigne, E François, R Faroux, D Smith, P Piedbois, H Naman, J Y Douillard, R Bugat

**Affiliations:** 1 Institut Claudius Regaud, 20-24 rue du Pont saint Pierre, Toulouse 31052, France; 2 Centre René Gauducheau, Boulevard Jacques Monod, Nantes 44805, France; 3 Clinique St Jean, 30 rue Bataille, Lyon 69008, France; 4 Hôpital Saint Joseph, 26 Boulevard Louvain, Marseille 13008, France; 5 Hôpital Civil, Le Parc, 46 rue du Stauffen, Colmar 68024, France; 6 Centre Léon Bérard, 28 rue Laënnec, Lyon 69373, France; 7 Centre Antoine Lacassagne, 33 avenue de Valombrose, Nice 06189, France; 8 Centre Hospitalier Départemental, rue des Oudairies, La Roche sur Yon 85925, France; 9 Centre Hospitalier Universitaire Saint André, 1 rue Jean Burguet, Bordeaux 33075, France; 10 Centre Hospitalier Universitaire Henri Mondor, 51 avenue du Maréchal de Lattre de Tassigny, Créteil 94000, France; 11 Centre Azuréen Cancérologie, 112 avenue du Docteur-Donat, Mougins 06250, France

**Keywords:** UFT, colorectal cancer, leucovorin, irinotecan, first-line treatment

## Abstract

This phase II trial was performed to evaluate the efficacy and tolerability of oral tegafur–uracil (UFT®) with leucovorin (LV) combined with intravenous (i.v.) irinotecan every 3 weeks (TEGAFIRI) as first-line treatment for patients with metastatic colorectal cancer (mCRC). Patients received oral UFT 250 mg m^−2^ day^−1^ and LV 90 mg day^−1^ in three divided daily doses for 14 days followed by a 1-week rest and i.v. irinotecan 250 mg m^−2^ as a 90-min infusion every 3 weeks. Tumour responses, assessed every two cycles using RECIST criteria, were reviewed by an independent review committee. In 52 evaluable patients, the best overall response rate was 33% (95% confidence intervals (CI) 20–47%; 1 complete and 16 partial responses). The median time to progression was 5.4 months (95% CI 3.02–7.52 months) and median overall survival was 14.9 months (11.73–17.97 months). A total of 307 cycles were administered, with a median number of five cycles per patient (range: 1–10). The most common grade 3/4 toxicities were neutropenia (25% of patients), diarrhoea (22%), vomiting (11%) and anaemia (11%). The TEGAFIRI regimen is a feasible, well-tolerated and convenient treatment option for patients with non-resectable mCRC.

The use of fluoropyrimidine therapy for patients with colorectal cancer (CRC) is now well established. 5-Fluorouracil (5-FU)-based chemotherapy has been the mainstay of treatment for advanced and metastatic colorectal cancer (mCRC) since its introduction in 1957. In recent years, enormous efforts have gone into improving its efficacy, including biochemical modulation and changing the method of its administration. The biomodulation of 5-FU by leucovorin (LV) has been shown to significantly improve response rates and prolong survival with high- and low-dose LV schedules (Bobbio-Pallavicini *et al*, 1993; Petrioli *et al*, 1995; Borner *et al*, 1998). Administration of 5-FU via continuous infusion has resulted in significantly better response rates when compared with bolus intravenous (i.v.) 5-FU, as demonstrated in a meta-analysis of six randomised studies (Meta-Analysis Group in Cancer, 1998), although only a modest survival benefit (12.1 *vs* 11.3 months; *P*<0.04) was demonstrated. The continuous infusion regimen was associated with a greater incidence of hand–foot syndrome (34 *vs* 13%), but the incidence of grade 3/4 neutropenia was significantly reduced.

In an attempt to improve the therapeutic index of 5-FU, research has also focussed on the study of 5-FU prodrugs and the selection of drugs with a better bioavailability profile, the aim being to increase tumour exposure and to reduce the complexity involved with continuous i.v. infusion administration. UFT is a combination of tegafur, an oral precursor of 5-FU, and uracil, a reversible dihydropyrimidine dehydrogenase (DPD) inhibitor. Tegafur is converted into 5-FU by the hepatic cytochrome *P*450 pathway (El Sayed and Sadee, 1982), whereas uracil enhances the half-life of converted 5-FU by competing for its degradation by DPD, this being the rate-limiting enzyme in the catabolism of 5-FU. Current clinical experience indicates that UFT with LV is a convenient, well-tolerated and effective alternative to i.v. 5-FU/LV for the treatment of advanced CRC. Two large, randomised phase III studies compared UFT plus LV given for 28 days every 35 days *vs* parenteral 5-FU/LV administered for 5 days every 28 or 35 days in previously untreated patients with mCRC (Carmichael *et al*, 2002; Douillard *et al*, 2002). In both studies, the oral and i.v. regimens resulted in comparable response rates and overall survival. However, substantial safety benefits were observed in the UFT plus LV group, with patients experiencing significantly less stomatitis/mucositis and myelosuppression, resulting in fewer episodes of febrile neutropenia.

In recent years, the introduction of chemotherapeutic agents, such as irinotecan, oxaliplatin and capecitabine, has led to significant advances in the treatment of patients with mCRC. The topoisomerase I inhibitor irinotecan was initially introduced as monotherapy for patients with mCRC refractory to 5-FU. In two randomised phase III trials, second-line irinotecan extended survival significantly when compared with supportive care (Cunningham *et al*, 1998) or 5-FU/LV infusion as second-line therapy (Rougier *et al*, 1998). In the first-line setting, the irinotecan plus 5-FU/LV combination produced better tumour response rates and longer progression-free and overall survival times than 5-FU/LV alone (bolus or infusion 5-FU regimens) in two large randomised clinical trials (Douillard *et al*, 2000; Saltz *et al*, 2000).

The convenient route of administration of UFT with LV combined with its efficacy and improved toxicity profile compared with i.v. 5-FU make it an attractive option for combining with irinotecan in the treatment of mCRC. The feasibility of this combination was evaluated in a phase I/II study performed in patients with advanced CRC (Price and Hill, 2000; Mackay *et al*, 2003). The recommended doses for further studies were UFT 250 mg m^−2^ day^−1^ and LV 90 mg day^−1^ given on days 1–14 with i.v. irinotecan 250 mg m^−2^ administered on day 1 every 3 weeks. The main dose-limiting toxicities were diarrhoea and febrile neutropenia (Mackay *et al*, 2003). Therefore, we initiated a phase II study using this dosage regimen to determine the efficacy and safety of the combination as first-line treatment for patients with mCRC.

## MATERIALS AND METHODS

### Patient selection

Patients with histologically or cytologically confirmed mCRC not suitable for curative surgery were included in this study. Prior major surgery, radiation and adjuvant or neoadjuvant chemotherapy had to be completed at least 4 weeks before study entry. Further eligibility criteria included male or female patients aged ⩾18 years; at least one uni- or bi-dimensionally measurable lesion; adequate bone marrow (absolute neutrophil count (ANC) ⩾1.5 × 10^9^ l^−1^, platelets >100 × 10^9^ l^−1^), liver (bilirubin ⩽1.5 × upper limit of normal (ULN) and transaminases ⩽2.5 × ULN or ⩽5 × ULN in case of hepatic metastasis) and kidney (serum creatinine ⩽1.5 × ULN) functions; Eastern Cooperative Oncology Group performance status of 0 or 1 and life expectancy ⩾12 weeks. Patients were excluded if they showed evidence of central nervous system metastases, serious intercurrent infections or concurrent active malignancies. Prior chemotherapy for metastatic disease was not permitted. Patients were excluded if prior radiation therapy had included a target lesion, unless the lesion was shown to have progressed after completion of radiation treatment or the patient had target lesions outside of any radiation ports. All patients provided written informed consent. The study was conducted in accordance with the Declaration of Helsinki Principle and Good Clinical Practice and was approved by an independent ethics committee.

### Treatment

UFT 250 mg m^−2^ day^−1^ and LV 90 mg day^−1^ were given together, in three divided daily doses, for 14 days (days 1–14), followed by a 1-week rest period; irinotecan 250 mg m^−2^ was administered as a 90-min i.v. infusion on day 1 of the 3-week cycle. No food could to be taken 1 h before or 1 h after each UFT dose. Duration of treatment was based on tumour response: patients with stable disease or a partial response (PR) could receive treatment until progression; those with a complete response (CR) could continue treatment for up to four cycles after documentation of CR.

Dosage modifications for both UFT and irinotecan were planned in the case of severe haematological and/or non-haematological toxicities.

During a treatment cycle, UFT/LV was withheld if a grade 4 haematological toxicity or a grade ⩾2 non-haematological toxicity developed (National Cancer Institute Common Toxicity Criteria (NCI-CTC), Version 2.0, 1998). In particular, patient with grade 2 diarrhoea were supposed to stop the treatment until toxicity resolved to baseline or grade ⩽1.

For the subsequent cycles, a maximum of 2 weeks for initiation of treatment was tolerated. Treatment was not resumed until haematological recovery (ANC ⩾1.5 × 10^9^ l^−1^ and platelets ⩾75 × 10^9^ l^−1^) and non-haematological toxicity resolved to baseline (except any grade of alopecia). Following grade 4 haematological toxicity, febrile neutropenia or grades 2–4 non-haematological toxicity (other than alopecia or fatigue), both the irinotecan and UFT doses were reduced by 50 mg m^−2^ in subsequent treatment cycles. Once the dose of UFT and/or irinotecan had been reduced, subsequent re-escalation of the dose was not permitted.

Concomitant treatment for diarrhoea consisted of loperamide as curative treatment. Standard anti-emetic therapy and premedication before the irinotecan infusion could be prescribed as required, at the discretion of the investigator. Haematopoietic colony-stimulating factors, antibiotics and erythropoietin could be administered prophylactically.

### Evaluation of tumour response and toxicity

Pretreatment evaluation included a complete history and clinical examination, haematological and biochemical profiles, electrocardiogram, chest X-ray and computerised tomography (CT) scan of the abdomen and other sites of disease when appropriate. During treatment, weekly complete blood cell count with differential and platelet count was obtained, with serum chemistry performed every 2 cycles. Performance status and physical examination were assessed before each cycle. Tumour assessment by CT scan was repeated every two cycles (6 weeks) and/or at the end of treatment, then every 3 months during follow-up using the same method as was used at baseline.

Response was evaluated according to Response Evaluation Criteria in Solid Tumours (Therasse *et al*, 2000). Complete response was defined as the disappearance of all clinical and radiological evidence of target lesions; PR as a ⩾30% decrease in the overall sum of the diameter of the target lesion(s); and progressive disease (PD) as a ⩾20% increase in the overall sum of the diameter of the target lesion(s). In case of PR or CR, a second assessment 4 weeks later was required for confirmation of response. All tumour measurements were reviewed by an independent review committee of radiologists. The duration of response was calculated from the date of the first treatment to the first date of documented progression for patients with PR and from the date of the occurrence of the CR to the first date of documented progression for patients with CR. Time to progression (TTP) was calculated as the time from the first day of treatment to the first date of documented progression or death. Overall survival was defined as the time from the first day of therapy (informed consent date) to the date of death.

Toxicity, graded according to the NCI-CTC (Version 2.0, 1998), was assessed by means of clinical and biological examinations before each cycle (weekly within a cycle for haematological toxicity), and at the end of treatment.

### Statistical analysis

This phase II study was designed using an exact single-stage procedure to detect a tumour response rate of at least 25% in patients with mCRC. Based on a significance level of 5% and a power of 90%, a minimum of 49 evaluable patients was to be included in the study. Assuming a 10% rate of non-evaluable patients, a total of 55 patients needed to be enrolled.

Analyses were performed using SAS® (Version 8.2; SAS Institute, Cary, NC, USA). Efficacy analyses were performed on both the intent-to-treat (ITT) population and the evaluable population, which was defined as all patients who received at least two cycles of treatment and had at least one tumour measurement. The safety analysis was performed in all patients who received at least one dose of study drugs. The primary end point of the study was the objective response rate (ORR), that is, the rate of CR plus PR. The ORR was computed with two-sided Clopper–Pearson confidence intervals (CI). Multivariate analysis was carried out using multiple logistic regressions to determine significant prognostic factors (among age, organ involved, previous adjuvant chemotherapy, performance status, lactate dehydrogenase and alkaline phosphatase at baseline); univariate analysis was entered into the model in single step (step method). Adjusted odds ratios and their 95% CI at the level of significance *P*<0.10 were provided.

**Figure 1:**
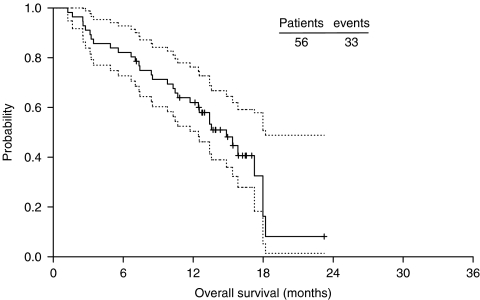
Kaplan–Meier curve of median survival.

Secondary efficacy criteria were duration of response, TTP and survival, which was estimated using the Kaplan–Meier product limit method, calculating 95% CI for median values (Figure 1). For toxicity analysis, the worst grade for each patient in all cycles of chemotherapy was used according to NCI-CTC criteria.

## RESULTS

From September 2002 to October 2003, 56 patients were included and treated in the study. Four patients were excluded from the efficacy analysis (three patients had no tumour evaluation after baseline and one patient had <2 treatment cycles).

### Patient characteristics

Patient and disease characteristics at inclusion are summarised in Table 1. More than half of the patients were male (54%) and the median age was 66 years (range: 42–88 years). The median time from initial diagnosis to study entry was 1.7 months (range: 0.2–86.2 months). Thirty-seven patients (66%) had synchronous metastases; 22 patients (39%) had ⩾2 metastatic sites with liver and/or lung as primary sites of metastases. Overall, 49 patients (88%) had received prior treatment for cancer, all of whom had surgery and 15 of whom (27%) had received adjuvant (*n*=13) or neoadjuvant (*n*=2) chemotherapy.

### Treatment exposure

Overall, 307 cycles were administered to 56 patients, with a median number of five cycles per patient (range: 1–10 cycles). The median duration of treatment was 17 weeks (range: 3–34 weeks). The mean (±s.d.) dose intensities of UFT (142.2±24.3 mg m^−2^ day^−1^) and irinotecan 76.8±7.6 mg m^−2^ week^−1^) corresponded to 85.3 and 92.2% of the scheduled doses, respectively. Treatment was delayed in 15% of cycles, mostly for reasons other than toxicity. The UFT and irinotecan doses were reduced in 4 and 5% of cycles, respectively, mainly as a result of grade 3 or 4 diarrhoea. Reasons for treatment discontinuation were: PD (*n*=23); investigator's decision (*n*=8); patient request (*n*=4); drug-related toxicity (*n*=4); symptomatic deterioration without objective evidence of progression (*n*=3); death (*n*=3) and other reasons (*n*=11).

### Independent review committee efficacy results

In the evaluable population (*n*=52), the ORR was 32.7%. (95% CI 20.0–47.0%), with one CR (1.9%) and 16 PR (30.8%). Stable disease was observed in 52% of patients. The ORR in the ITT population (*n*=56) was 30.4% (95% CI, 19.0–44.0%; Table 2). Median response duration was 7.7 months (95% CI 56.0–9.5 months), median TTP was 5.4 months (95% CI 3.0–7.5 months); and median survival was 14.9 months (95% CI 11.7–18.0 months).

In the multivariate analysis, only alkaline phosphatase grade at baseline was an independent prognostic factor of the objective response (odd ratio=0.389 (0.148–1.020)); the lower the grade of alkaline phosphatase at baseline, the higher the ORR.

### Safety results

As expected, myelosuppression and gastrointestinal disorders were the most commonly observed toxicities resulting from study treatment (Table 3). Grade 3/4 neutropenia occurred in 25% of patients and 8% of cycles (grade 4 toxicity in 13% of patients). Only one patient experienced febrile neutropenia, which lasted for 5 days. Grade 3/4 anaemia occurred in 11% of patients and 2% of cycles (one patient had grade 4 toxicity). Grade 4 thrombocytopenia (no grade 3 thrombocytopenia was observed) occurred in two patients (4%) and in 1% of cycles, but was not complicated by haemorrhage.

Grade 3/4 non-haematological toxicities related to study drugs consisted mainly of diarrhoea (23% of patients; 5% of cycles), vomiting (11% of patients; 3% of cycles), fatigue (7% of patients; 2% of cycles) and nausea (7% of patients; 2% of cycles) (Table 3). Only two patients experienced a grade 4 adverse event related to the study drugs (diarrhoea and fatigue). Few patients experienced adverse events leading to hospitalisation: grade 4 asthenia (one patient), grade 3 dyspnoea (one patient), grade 4 mediastinitis (one patient), all of which were systematically related, in the investigator's opinion, to PD. There were no treatment-related deaths during the study. No hand–foot syndrome greater than grade 2 was observed.

## DISCUSSION

Colorectal cancer is a common disease that is difficult to treat effectively and safely. Chemotherapy options are relatively limited at present and more effective, well-tolerated treatments are urgently needed to combat the considerable mortality and morbidity associated with the disease. Given the limitations of available chemotherapies, the tumour response rate of 33%, disease stabilisation rate of 52% and the favourable tolerability profile observed in this study of UFT with LV plus irinotecan is encouraging, worthwhile and comparable with response rates achieved with 5-FU–irinotecan combination regimens.

Combinations of 5-FU/LV with irinotecan (FOLFIRI, AIO or IFL regimens) or oxaliplatin (FOLFOX4 or FOLFOX6 regimens) are considered to be standard first-line treatments for patients with mCRC (Saltz *et al*, 2001; Meyerhardt and Mayer, 2005). Irinotecan has been evaluated in combination with 5-FU/LV in two large multicentre phase III trials using either the bolus 5-FU schedule (Mayo Clinic regimen, *n*=683) in North America (Saltz *et al*, 2000) or a 48-h infusion programme in Europe (*n*=385) (Douillard *et al*, 2000). The response rates observed in those studies (39 and 35%, respectively) are slightly higher than that obtained in the present study (33%); however, the median overall survival times reported by Saltz *et al* (14.8 months) and Douillard *et al* (17.4 months) are comparable with the 14.9 months obtained in the present study.

The efficacy of the TEGAFIRI regimen is superior to that of UFT with LV alone and higher than the 21% response rate reported by Mendez *et al* in a phase II study of weekly irinotecan combined with UFT plus LV in the first-line setting (Mendez *et al*, 2005). Interesting, results were obtained with a regimen in which UFT with LV plus oxaliplatin was alternated with UFT with LV plus irinotecan in 41 patients with mCRC (Petrioli *et al*, 2004). In that study, an ORR of 58.5%, median overall survival of 17.3 months and median TTP of 8.8 months were reported. Similarly, Sheikh *et al* reported promising results in a phase I study of alternating UFT–oxaliplatin and UFT–irinotecan in the first-line treatment of patients with mCRC (Sheikh *et al*, 2007). A response rate of 71% was reported in 25 patients, with a median TTP of 8.8 months. UFT with LV has also been evaluated in combination with oxaliplatin (the TEGAFOX regimen) using a similar 3-weekly oxaliplatin schedule (Bennouna *et al*, 2006). The TEGAFOX regimen provided similar efficacy to that of the TEGAFIRI regimen, with an ORR of 34% in 58 patients and a median TTP and survival of 5.9 and 18.2 months, respectively.

The adverse event profile of the TEGAFIRI regimen was acceptable and consistent with results from a phase I trial in which grade 3/4 diarrhoea and febrile neutropenia were dose limiting (Mackay *et al*, 2003). Most haematological and non-haematological adverse events in the present study were mild or moderate in intensity. The most frequent grade 3/4 adverse events were neutropenia and diarrhoea, which were seen in 25 and 23% of patients, respectively. Grade 4 toxicity was infrequent and only one patient had febrile neutropenia. As might be expected, the addition of irinotecan to the UFT regimen was associated with a modest increase in adverse events *vs* UFT with LV. In the studies by Douillard *et al* (2002) and Carmichael *et al* (2002), the incidence of grade 3/4 neutropenia in UFT-treated patients (3% of patients in both studies) was lower than that observed in the present study (25% of patients). However, the incidences of grade 3/4 diarrhoea appeared similar (21 and 18% in the Douillard and Carmichael studies, respectively, *vs* 22% in the present study). While comparisons between clinical studies are made with caution, grade 3/4 neutropenia appeared to be less common with the TEGAFIRI regimen compared with the FOLFIRI combination, occurring in 25% of TEGAFIRI patients *vs* 54 and 46% of patients treated with bolus or infusional 5-FU/LV in combination with irinotecan, respectively (Douillard *et al*, 2000; Saltz *et al*, 2000). Only one patient (2%) in the present study experienced febrile neutropenia, compared with 7 and 3% of patients in the Douillard and Saltz studies, respectively (Douillard *et al*, 2000; Saltz *et al*, 2000). The incidence of grade 3/4 diarrhoea in these two phase III trials was 23 and 14%, respectively. In the weekly irinotecan plus UFT with LV regimen, the most frequently reported grade 3/4 toxicities were neutropenia (11% of patients) and delayed diarrhoea (28%) (Mendez *et al*, 2005).

UFT with LV treatment is a convenient, well-tolerated and effective alternative to i.v. 5-FU/LV. A crossover study (*n*=36) evaluating patient preference for UFT with LV or i.v. 5-FU/LV chemotherapy in mCRC showed that 84% of patients preferred oral treatment over i.v. treatment (Borner *et al*, 2002). UFT may be administered without visits to the hospital, resulting in reduced treatment costs (Murad *et al*, 1997) and increased convenience for the patient. The oral schedule also allows patients to temporarily withhold a dose of the drug if adverse events emerge, for example grade 2 diarrhoea, thereby preventing the toxicity from becoming severe. Patients can then restart the medication after 1–2 days, when the diarrhoea resolves (Hoff and Pazdur, 1998). Simply eliminating several doses at the onset of early-stage diarrhoea can prevent its progression to a serious or even life-threatening toxicity that might otherwise require hospitalisation.

In conclusion, this phase II study has added to the clinical evidence that UFT with LV is a good combination partner for irinotecan in the treatment of patients with non-resectable mCRC. The results from this study suggest that administration of irinotecan every 3 weeks plus daily UFT with LV is a feasible, well-tolerated and convenient treatment option for patients with this condition.

## Figures and Tables

**Table 1:** Patients and disease characteristics at baseline (*n*=56)

**Characteristic**	**No. of patients**	**%**
*Sex*		
Male	30	54
Female	26	46
Median age (range, years)	66.0 (42–88)	
		
*ECOG performance status*		
0	32	57
1	23	41
Missing	1	2
		
*Primary site*		
Colon	31	55
Rectum	17	30
Colorectal	7	13
Missing	1	2
		
*Number of organs involved*		
1	34	61
2	16	29
3	5	9
4	1	2
		
*Organ involvement*		
Liver only	25	45
Lung only	3	5
Liver and lung	14	25
Lymph nodes	4	7
Peritoneum	2	4
		
*Prior therapy*		
Surgery	49	88
Radiotherapy	9	16
Adjuvant/neoadjuvant chemotherapy	15	27

ECOG=Eastern Cooperative Oncology Group.

**Table 2:** Efficacy results based on IRC assessment in the ITT population (*n*=56)

**Outcome**	**Value**
*Best tumour response, n* (%)	
Complete response	1 (2)
Partial response	16 (29)
Stable disease	27 (48)
Progressive disease	5 (9)
Not evaluable	7 (13)
Overall best response rate (95% CI)	30.4 (19.0–44.0)
Median duration of response (95% CI, months)	7.69 (5.95–9.46)
Median time to progression (95% CI, months)	5.45 (3.02–7.52)
Median survival time (95% CI, months)	14.85 (11.73–17.97)

CI=confidence interval; IRC=independent review committee; ITT=intent-to-treat.

**Table 3:** Adverse events related to study drugs reported in at least 5% of patients according to NCI-CTC criteria

	**By patient (*n*=56)**		**By cycle (*n*=307)**	
**Toxicity, *n* (%)**	**All grades**	**Grade 3/4**	**All grades**	**Grade 3/4**
*Haematological*				
Leucopoenia	36 (64)	6 (11)	111 (36)	13 (4)
Neutropenia	35 (63)	14 (25)	121 (39)	26 (8)
Anaemia	37 (66)	6 (11)	124 (40)	7 (2)
Thrombocytopenia	5 (9)	2 (4)	5 (2)	2 (1)
				
*Non-haematological*				
Nausea	44 (79)	4 (7)	136 (44)	7 (2)
Diarrhoea	42 (75)	13 (23)	141 (46)	15 (5)
Fatigue	33 (59)	4 (7)	86 (28)	7 (2)
Alopecia	33 (59)	NA	202 (66)	NA
Vomiting	30 (54)	6 (11)	94 (31)	9 (3)
Abdominal pain	19 (34)	1 (2)	40 (13)	1 (<0.5)
Anorexia	10 (18)	1 (2)	13 (4)	1 (<0.5)
Constipation	6 (11)	1 (2)	7 (2)	1 (<0.5)
Pyrexia	6 (11)	0	7 (2)	0
Headache	6 (11)	0	14 (4)	0
Asthenia	4 (7)	2 (4)	15 (5)	4 (1)
Cholinergic syndrome	4 (7)	1 (2)	5 (2)	2 (1)
Weight decreased	5 (9)	0	17 (6)	0
Paraesthesia	3 (5)	0	4 (1)	0
Vertigo	3 (5)	0	4 (1)	0

NA=not applicable; NCI-CTC=National Cancer Institute Common Toxicity Criteria.
